# Target Detection Based on Improved Hausdorff Distance Matching Algorithm for Millimeter-Wave Radar and Video Fusion

**DOI:** 10.3390/s22124562

**Published:** 2022-06-17

**Authors:** Dongpo Xu, Yunqing Liu, Qian Wang, Liang Wang, Renjun Liu

**Affiliations:** 1 School of Electronics and Information Engineering, Changchun University of Science and Technology, Changchun 130022, China; 2019200085@mails.cust.edu.cn (D.X.); 2019100528@mails.cust.edu.cn (Q.W.); 2020100646@mails.cust.edu.cn (R.L.); 2 Intelligent Perception and Processing Technology Laboratory, Beijing 100124, China; wangliang002@cust.edu.cn

**Keywords:** intelligent transportation systems, millimeter-wave radar, video, spatio-temporal alignment, target matching

## Abstract

The intelligent transportation system (ITS) is inseparable from people’s lives, and the development of artificial intelligence has made intelligent video surveillance systems more widely used. In practical traffic scenarios, the detection and tracking of vehicle targets is an important core aspect of intelligent surveillance systems and has become a hot topic of research today. However, in practical applications, there is a wide variety of targets and often interference factors such as occlusion, while a single sensor is unable to collect a wealth of information. In this paper, we propose an improved data matching method to fuse the video information obtained from the camera with the millimetre-wave radar information for the alignment and correlation of multi-target data in the spatial dimension, in order to address the problem of poor recognition alignment caused by mutual occlusion between vehicles and external environmental disturbances in intelligent transportation systems. The spatio-temporal alignment of the two sensors is first performed to determine the conversion relationship between the radar and pixel coordinate systems, and the calibration on the timeline is performed by Lagrangian interpolation. An improved Hausdorff distance matching algorithm is proposed for the data dimension to calculate the similarity between the data collected by the two sensors, to determine whether they are state descriptions of the same target, and to match the data with high similarity to delineate the region of interest (ROI) for target vehicle detection.

## 1. Introduction

An intelligent transportation system (ITS) [1] is a management system that uses advanced information technology, information and communication technology, and modern management ideas and methods to achieve coordinated operation and interconnection between various transportation elements, such as people, vehicles, and roads. The traditional traffic monitoring system mainly consists of video monitoring technology, which can only simply achieve the real-time requirements of the monitoring system; when a large amount of data exists, it is unable to process them efficiently and accurately to meet the hardware and software requirements. The system monitoring process to obtain the vehicle often comes from the monitoring video, but due to the characteristics of the video itself, the actual scene is easily obscured, due to foggy weather and light changes and other interference, but also due to hardware failure or thunderstorm weather, making the video detection of vehicles exceptionally difficult. Therefore, the study of how to use multi-sensor data to improve the recognition rate of vehicle tracking in complex backgrounds has become an important topic in the field of intelligent transportation systems today [2]. A comparison of the performance of each sensor is shown in Table 1. Millimeter-wave radar sensors can accurately perform position and velocity estimation of targets, but cannot accurately acquire target characteristics; video information can clearly achieve the feature extraction of targets, but, due to its principle characteristics, cannot accurately calculate the movement speed and position. Although each sensor has its own characteristics, they all share a common drawback: during feature extraction, the target’s characteristic information, such as license plate number and car model, is often missing. A detection system that uses a fusion of radar and camera sensors can combine their detection advantages to obtain the feature information, position, and velocity of the target simultaneously, and has high real-time performance with low hardware requirements and a broader range of applications [3].

Millimeter-wave radar works in the 30–300 GHZ band, with a detection range of 250 m and a detection angle of 30 degrees, and has been widely used at home and abroad due to its strong penetration, good weather resistance, and small size. However, the low power and high transmission loss of millimeter-wave radar lead to unsatisfactory detection results in long-distance detection, and millimeter-wave radar will also be subject to electromagnetic interference in operation, limiting the development of millimeter-wave technology in the field of communication. Millimeter-wave radar is widely used in the field of intelligent driving, as a vehicle radar for front vehicle detection and collision prevention, but it is rarely involved in areas such as intelligent transportation systems, so the study of millimeter-wave radar in the transportation system for vehicle detection technology has important scientific significance.

In practical application scenarios, the use of multi-sensor fusion for target identification and tracking can achieve results that cannot be obtained by a single sensor, improving the accuracy of target detection [4]. Cameras measure a large range of data but are susceptible to environmental influences; LIDAR has a very accurate perception of the environment; and millimeter-wave radar can obtain information about the target’s motion state and improve the overall detection effect. Common sensor combinations are LIDAR and camera fusion [5] and the fusion of infrared sensors and cameras; based on considerations of actual traffic monitoring costs, a combination of millimeter-wave radar and cameras can be used, using millimeter-wave radar to obtain information on target dynamics and combining it with the rich information obtained by the camera to meet the requirements for target vehicle detection, while covering all the advantages of single sensor detection. Millimeter-wave radar is highly robust in bad weather and can be combined with machine vision to be able to improve the detection accuracy. Researchers Ji Z et al. [6], from the Toyota Technology Centre in Japan, have developed an obstacle detection and classification system combining millimeter-wave radar with a camera. The system uses images taken by the camera to delineate the range of interest in the images detected by the millimeter-wave radar, and then uses a neural network to rapidly identify the detected vehicles, but the detection accuracy of the method is not high. To address this problem, Anselm et al. turned to the adaboost machine learning algorithm, which is commonly used for face detection, to detect vehicles, with greatly improved robustness and throughput [7]. The above fusion research on radar and cameras focuses on spatial coordinate matching, mainly for the projection of the area of interest of the radar to the camera area, but less work has been done on data matching. Tao uses a combination of vehicle-mounted radar and monocular vision to improve the probability of obstacle detection [8]. This method uses radar to roughly select obstacles, and then uses video to detect shadow areas to check obstacles and false alarms, reducing the influence of false alarm obstacles. However, the Euclidean distance matching method is too one-sided when applied to complex vehicle conditions, and it is likely to cause a mismatch. Some types of noise have too much influence on the target.

Based on this, this paper proposes an improved Hausdorff distance matching method, aiming at the matching problem of source data in the millimeter-wave radar and video fusion detection model; here, the data after spatial coordinate transformation are registered, and this is compared with the traditional Euclidean distance matching method. Compared with the Hausdorff matching method, it not only addresses the shortcomings of the Euclidean distance, but also uses the velocity component as the weight to increase the accuracy of the Hausdorff matching. By calculating the similarity and deviation values between the data obtained by the two sensors, data with a high degree of similarity are targeted. The matching algorithm is a modified Hausdorff distance matching algorithm, and by calculating the similarity and deviation values between the data obtained by the two sensors, the data with a high degree of similarity will be target matched. Finally, the successfully matched target data are used to characterize the motion information of the object, to achieve multi-sensor fusion target detection, and to compare the single sensor detection and fusion detection results.

## 2. Spatio-Temporal Calibration of Sensors

Before fusing the information from two different sensors, the target data measured by these two sensors need to be converted to the same reference coordinate system by joint calibration, and the sensors need to be aligned in the time dimension. In recent years, more and more scholars have invested more effort in studying the alignment of radar and camera, but most of these alignments have focused on the joint calibration of on-board radar and cameras, so most of the alignments of radar and cameras involve the alignment of LIDAR and cameras [9,10]. However, millimeter-wave radar and LIDAR work with large differences in performance and do not acquire the same type of target data, so this calibration method is not suitable for the calibration of millimeter-wave radar and camera. Foreign scholars Sugimoto S et al. combined the imaging principle of the camera and established a camera-based joint calibration model for millimeter-wave radar and camera [11]. Some scholars also ignore the influence of the internal and external parameters of the camera on the spatial conversion results and directly map the millimeter-wave radar data into the pixel coordinate system of the image to form the radar detection region of interest; this method completes the conversion of the coordinate system between the millimeter-wave radar and the camera, but the detection of the target vehicle is not effective [12]. Combined with the calibration methods in current multi-sensor fusion research, the joint radar–camera calibration method used in this paper is as follows: the spatial conversion is achieved by establishing a spatial coordinate conversion model for both radar and camera sensors, unifying both radar and video data into the image pixel coordinate system, and then the Zhang Zhengyou camera calibration method [13,14] is used to obtain the internal and external camera parameters to obtain the complete spatial conversion matrix relationship, and, finally, the two sensors are calibrated in the time dimension using Lagrangian interpolation, so that the data measured by the two sensors are collected under the same moment.

### 2.1. Spatial Calibration

**Figure 1:**
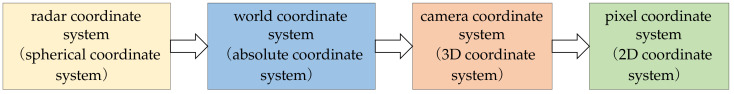
Spatial calibration process for radar and camera.

The millimeter-wave radar and the camera belong to two different types of environmental measurement sensors. When the two sensors collect data from the target object, they obtain different types of target data and the coordinate systems that characterize the information are independent of each other. The millimeter-wave radar operates in the spherical coordinate system, the camera operates in the camera coordinate system, while the video image operates in the image coordinate system; the conversion relationship between the coordinate systems is shown in Figure 1.

**Figure 2:**
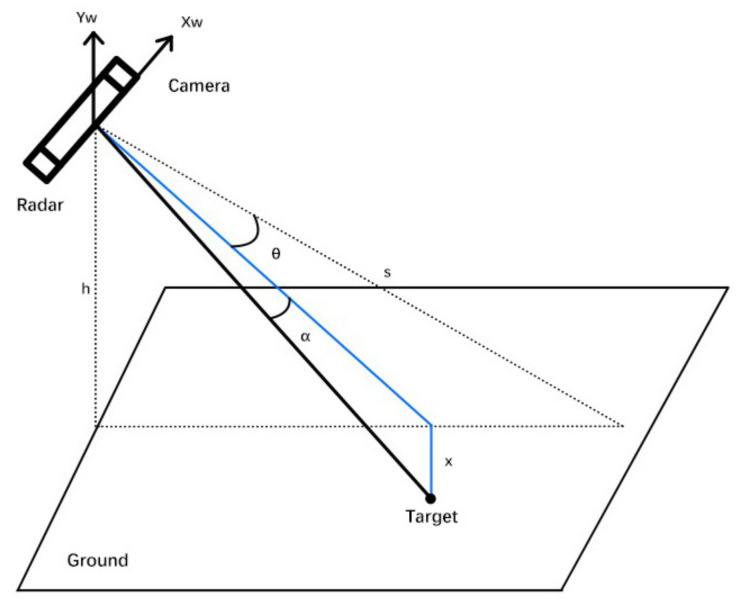
Schematic diagram of the conversion of radar data from the radar coordinate system to the world coordinate system.

The target data collected by the radar are first transformed from the spherical coordinate system in which the radar is located into the world coordinate system, which is a three-dimensional Cartesian coordinate system. When actually installed, the two sensors are physically very close to each other and can be approximated at the same position, so the spatial position where the radar and camera are located can be treated as having the same coordinates. The process of quasi-transforming the millimeter-wave radar coordinate system to the world coordinate system is shown in Figure 2.

The radar sensor can obtain information on the distance, velocity, and azimuth of the target, as shown in Figure 2. Assuming that the distance between the target and the sensor is *r* and the azimuth angle α, then the angular coordinate conversion relationship yields the components of the radar-detected target on both axes in the world coordinate system as follows.

(1) x=rsin(α)

(2) y=rcos(α)

For the millimeter-wave radar, the $O-X_{w}Y_{w}Z_{w}$ three-dimensional coordinate system is established, assuming that the height and pitch angle of the radar installation are *h* and θ, respectively, the distance between the radar sensor and the extension of its emitted signal and the ground intersection is defined as *s*, the extension of *s* is the $O-Z_{w}$ positive direction, the left direction of the sensor plane is the $O-X_{w}$ positive direction, and the vertical upward direction is the $O-Y_{w}$ positive direction.

The established coordinate system is analyzed and the corresponding conversion relationship between the target position and the radar position is obtained in $O-X_{w}Y_{w}Z_{w}$ by means of Equations (1) and (2).

(3) $$X_{w}=-x$$

(4) $$Y_{w}=-y\sin\left(\theta \right)$$

(5) $$Z_{w}=y\cos\left(\theta \right)$$

based on the geometric relationships depicted in Figure 2, it can be seen that

(6) $$\theta =\arccos\left(\frac{h}{s}\right)-\arccos\left(\frac{h}{y}\right)$$

**Figure 3:**
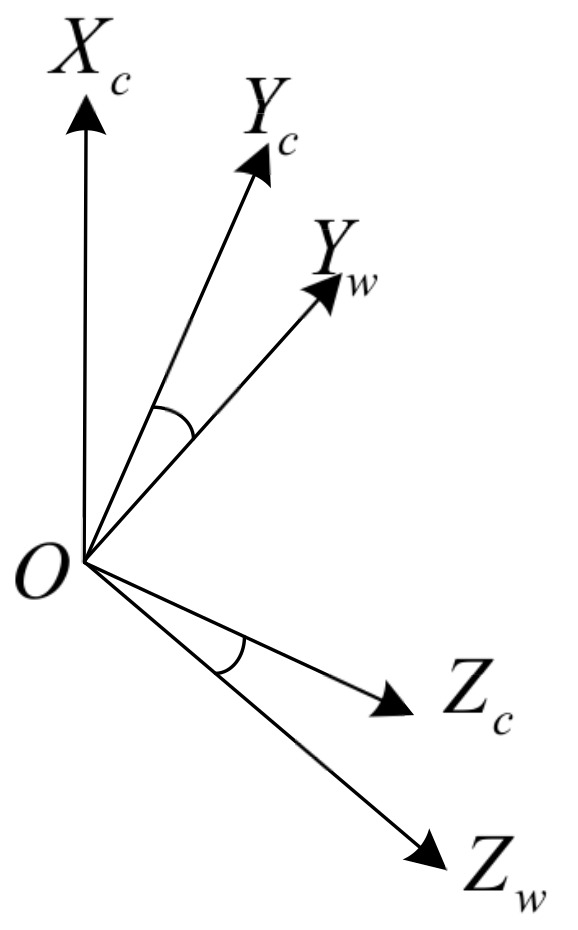
Diagram of the conversion of the world coordinate system to the camera coordinate system.

In a real situation, the mounting positions of the millimeter-wave radar and the camera can be approximated as the same position, depending on the spatial scale. Ignoring the error in the origin of the two coordinate systems, the world coordinate system of the radar is rotated so that it corresponds to the camera coordinate system. In Figure 3, $O-X_{w}Y_{w}Z_{w}$ is the radar world coordinate system and $O-X_{c}Y_{c}Z_{c}$ is the camera world coordinate system. Assuming that the O−YZ planes of the two coordinate systems have been completely overlapped after two rotations, the two coordinate systems can be completely overlapped by rotating the radar world coordinate system counterclockwise around the $O-X_{w}$-axis by an angle of γ.

The rotational transformation shown in Figure 3 can be written in the following form:

(7) $$X_{c}=X_{w}$$

(8) $$Y_{c}=Y_{w}\cos\left(\gamma \right)+Z_{w}\sin\left(\gamma \right)$$

(9) $$Z_{c}=Z_{w}\cos\left(\gamma \right)+Y_{w}\sin\left(\gamma \right)$$

where γ is the Euler angle, i.e., the angle mapped from the real coordinate system to the camera coordinate system by transformation. Since the camera and the millimeter-wave radar can be mounted in almost the same world coordinate system, the camera mounting height *h* from the ground determines the amount of translation during the transformation, and the rotation matrix is uniquely related to the γ angle, so the external parameters of the camera depend on the mounting height *h* and the rotation angle γ.

The conversion from the coordinate system $O-X_{w}Y_{w}Z_{w}$ to the coordinate system $O-X_{c}Y_{c}Z_{c}$ is based on the same principle as the single conversion, and the conversion relationship can be completed by multiple conversions, which are not repeated here.

**Figure 4:**
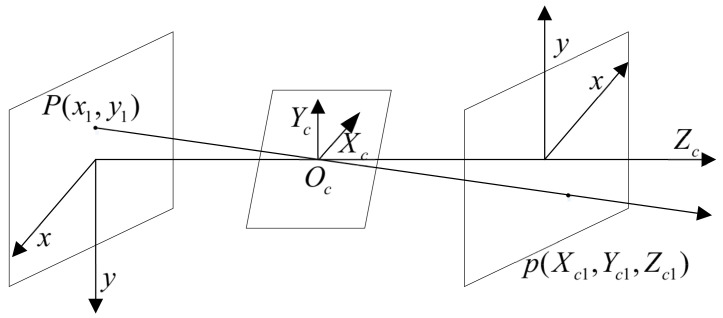
Diagram of the conversion of radar data from the camera coordinate system to the image coordinate system.

After the radar data have been converted into the corresponding coordinate system, they are further transformed into the coordinate system O−xy in the corresponding image by means of the camera’s small-aperture imaging principle. The transformation of the image coordinate system can be realized by several basic image processing methods. The conversion between the image coordinate system and the radar coordinate system is facilitated by the small-aperture imaging model. The method is simple, intuitive, and easy to implement programmatically, through simulation experiments and error analysis. The results show that the method is feasible and effective. The derivation process is shown in Figure 4.

A point $p(X_{c},Y_{c},Z_{c})$ in a camera with focal length *f* is imaged and projected onto a point $p^{\prime }\left(x,y\right)$ on the frame image, according to the geometric relationship.

(10) $$x=\frac{X_{c}}{Z_{c}}f$$

(11) $$y=\frac{Y_{c}}{Z_{c}}f$$

the world coordinate system is converted to a pixel coordinate system as follows:

(12) $$\begin{array}{l} Z_{c}\left[\begin{array}{c} u\\ v\\ 1 \end{array}\right]=\left[\begin{array}{ccc} 1/d_{x} & 0 & u_{0}\\ 0 & 1/d_{y} & v_{0}\\ 0 & 0 & 1 \end{array}\right]\left[\begin{array}{c} f\;0\;0\;0\\ 0\;f\;0\;0\\ 0\;0\;1\;0 \end{array}\right]\left[\begin{array}{c} R\;\;\;T\\ O^{T}\;1 \end{array}\right]\left[\begin{array}{c} X_{w}\\ Y_{w}\\ Z_{w}\\ 1 \end{array}\right]\\ =\left[\begin{array}{llll} a_{x} & 0 & u_{0} & 0\\ 0 & a_{y} & v_{0} & 0\\ 0 & 0 & 1 & 0 \end{array}\right]\left[\begin{array}{c} R\;\;\;T\\ O^{T}\;1 \end{array}\right]\left[\begin{array}{c} X_{w}\\ Y_{w}\\ Z_{w}\\ 1 \end{array}\right]\\ =M_{2}M_{1}\left[\begin{array}{l} X_{w}\\ Y_{w}\\ Z_{w}\\ 1 \end{array}\right]=M\left[\begin{array}{l} X_{w}\\ Y_{w}\\ Z_{w}\\ 1 \end{array}\right] \end{array}$$

where $a_{x}=\frac{f}{d_{x}}$, $a_{y}=\frac{f}{d_{y}}$, matrix $M_{1}$ are external camera parameters, consisting of a rotation matrix and a translation vector; matrix $M_{2}$ is an internal camera parameter, and the value of $M_{2}$ depends on $a_{x},a_{y},u_{0},v_{0}$. A point in space can be transformed into the image plane coordinate system by means of Equation (11).

The correspondence of point $P(X_{r},Y_{r})$ in the radar coordinate system mapped to point $P(X_{c},Y_{c},Z_{c})$ in the camera coordinate system is shown in Equation (13).

(13) $$\left\{\begin{array}{c} X_{c}=-Y_{r}\\ Y_{c}=H_{0}\\ Z_{c}=X_{r} \end{array}\right.$$

using the above equation, the transformation matrix for converting the millimeter-wave radar coordinate system to the image pixel coordinate system is derived under ideal conditions as follows:

(14) $$\left[\begin{array}{c} x\\ y\\ 1 \end{array}\right]=\left[\begin{array}{c} f\;0\;0\\ 0\;f\;0\\ 0\;0\;1 \end{array}\right]\left[\begin{array}{c} X_{t}\\ Y_{t}\\ 1 \end{array}\right]$$

where $X_{t}=\frac{X_{c}}{Z_{c}}$, $Y_{t}=\frac{Y_{c}}{Z_{c}}$.

It follows that the internal parameters of the camera depend on the focal length of the camera.

### 2.2. Timeline Calibration

**Figure 5:**
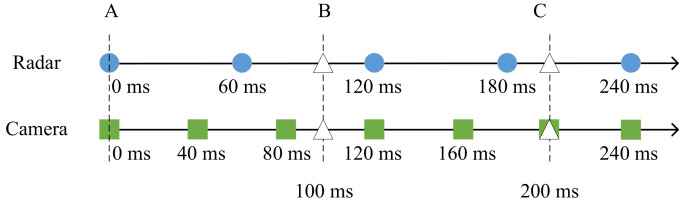
Sensor time alignment.

The interpolation and extrapolation method of temporal alignment is used to interpolate and extrapolate the measurement data of different sensors within a certain time period to predict the detection values of each sensor within the measurement time period. The algorithm allows multiple sensors to collect information on the same target, obtain the status detection results of each sensor on the target at the current moment, and use the information to calculate the relative relationship between the sensors at the next moment. The average sampling cycle period of the millimeter-wave radar selected in this paper is 60 ms, and the average sampling cycle of the camera is around 40 ms. The Lagrangian interpolation method is used for the temporal alignment of the two sensors, and the specific steps are as follows: assuming the sampling cycle of the fusion system T=100 ms, the measured values of the sensors are predicted separately, the interpolation process is performed within the fusion cycle, the interpolated data for each cycle sampling time point are extracted, and these data are used as an approximation of the actual radar and camera measurements for the next fusion step. A schematic diagram of the temporal alignment is shown in Figure 5.

In the time alignment diagram shown in Figure 5, the blue circles are the sampling moments of the millimeter-wave radar sensor, the green squares are the sampling moments of the camera, and the three moments *A*, *B*, and *C* are the sampling moments of the radar and camera multi-sensor fusion system, 0 ms, 100 ms, and 200 ms, respectively.

**Figure 6:**
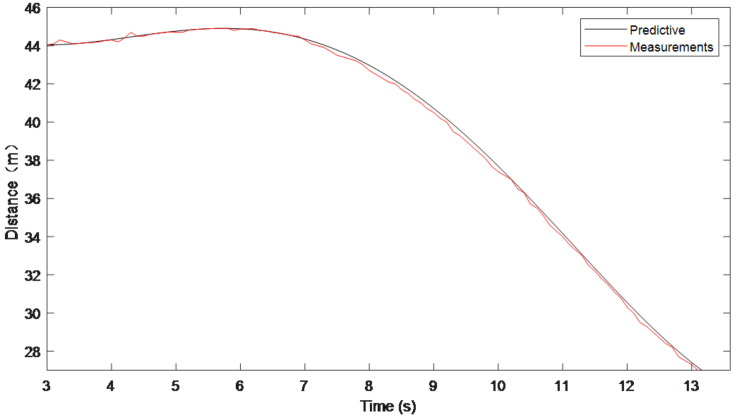
Sensor data time synchronization.

The data from the two sensors at time *A* do not need to be aligned. At moments *B* and *C*, however, there are deviations in the sampling times of the two sensors, and the sensor observations must be predicted. In this paper, the measurements are predicted using the state prediction method for the 60 ms and 120 ms sampling moments of the radar sensors, respectively, and the predicted states are interpolated with the actual radar measurements at these two moments to obtain an interpolated approximation, so that the approximation at moment *B* can be solved. The same interpolation method is used to estimate the interpolated values of the moments using the measured values of the radar at 180 ms and 240 ms. The interpolation function is shown in Equation (15).

(15) $$\begin{array}{c} x\left(t\right)=\frac{\left(t-t_{k}\right)\left(t-t_{k+1}\right)}{\left(t_{k-1}-t_{k}\right)\left(t_{k-1}-t_{k+1}\right)}x_{k-1}+\frac{\left(t-t_{k-1}\right)\left(t-t_{k+1}\right)}{\left(t_{k}-t_{k-1}\right)\left(t_{k}-t_{k+1}\right)}x_{k}\\ +\frac{\left(t-t_{k-1}\right)\left(t-t_{k}\right)}{\left(t_{k+1}-t_{k-1}\right)\left(t_{k+1}-t_{k}\right)}x_{k+1} \end{array}$$

using the Lagrangian interpolation method described above to time-calibrate the distance traveled by the target vehicle measured by the camera, the predicted and actual measured values were obtained and are shown in Figure 6.

As can be seen from the time synchronization results shown in Figure 6, the actual measured target distance from the camera is predicted by the Lagrangian interpolation method and the distance prediction obtained deviates very little from the actual value. The experimental results show that the time calibration method proposed in the paper works well.

## 3. Proposed Fusion Detection Algorithm

After the spatio-temporal alignment of the target data collected by the millimeter-wave radar and the camera is completed, the radar data are spatially transformed into the image coordinate system to form the radar target of interest on the image, but this does not identify whether the target is the same vehicle target. The differences in them have a significant impact on the fusion detection system degree, so it is necessary to calculate the detection performance of each sensor. Their detection ranges vary, and only a small amount of data and information describing the same target object is detected by both sensors; in addition, most of the measured data from the radar and camera are independent of each device’s target measurements, and the literature refers to these disconnected data as pseudo-point pairs [15], which, if not removed, reduce the degree of similarity between targets. Thus, we match the points with the greatest similarity according to the corresponding criterion.

### 3.1. Target Matching

The main traditional multi-sensor data matching algorithms, in general, are region of interest matching [16,17] and the distance sorting matching method. Due to the complexity of the region matching method algorithm, it leads to the detection algorithm taking a longer time in practical application, which is not conducive to real-time detection. For the distance-ordered matching method, Besl et al. [18] proposed an iterative nearest point matching algorithm (referred to as the ICP algorithm), in which the target data detected by the millimeter-wave radar are transformed through a series of coordinates in space to form a radar region of interest in the image, which is represented by a point set *P*. The Faster R-CNN algorithm is then used to detect the target object in the image and the target edge of the center point under the rectangular box to form a point set *Q*. The purpose of the Euclidean distance method is to represent the distance between two points in space by calculating and ordering the distance between each point in point set *P* and the corresponding point in point set *Q*. Artifacts are eliminated according to the overlap ratio between the two point sets, which improves the algorithm’s operational efficiency [19]. This target matching algorithm is relatively simple to implement after using Euclidean distance sorting, but because the method only calculates the absolute distance in space between two points, without considering the connection between various characteristics, and the matching points in this paper originate from two different sensors, the Euclidean distance matching method is not ideal to obtain matching results and the error is relatively large. In summary, based on the fact that both millimeter-wave radar and video can measure and calculate the distance and velocity values of the detected target, this paper proposes an improved Hausdorff distance matching algorithm, which is based on Hausdorff distance matching with weighted target velocity information integrated so as to determine whether the target data measured by the two sensors are for the same moving vehicle.

#### 3.1.1. Hausdorff Distance Matching Algorithm

The Hausdorff distance, or Hausdorff metric, is the distance between two subsets of a metric space, which can be transformed from a non-empty subset of the metric space itself into a metric space [20]. Informally, if every point of a set is infinitely close to a point of another set, then the two sets can be described as close in Hausdorff distance, which is simply “the maximum distance from one set to the nearest point in the other set”. More formally, the Hausdorff distance from a set *A* to a set *B* is a very large minuscule function defined as a directed Hausdorff distance function.

(16) $$h\left(A,B\right)=\max_{a\in A}\left\{\min_{b\in B}\left\{d\left(a,b\right)\right\}\right\}$$

where *a* and *b* are the points of the sets *A* and *B*, respectively, and d(A,B) is any measure between these points; for simplicity, we will take d(A,B) to be the Euclidean distance between *A* and *B*.

**Figure 7:**
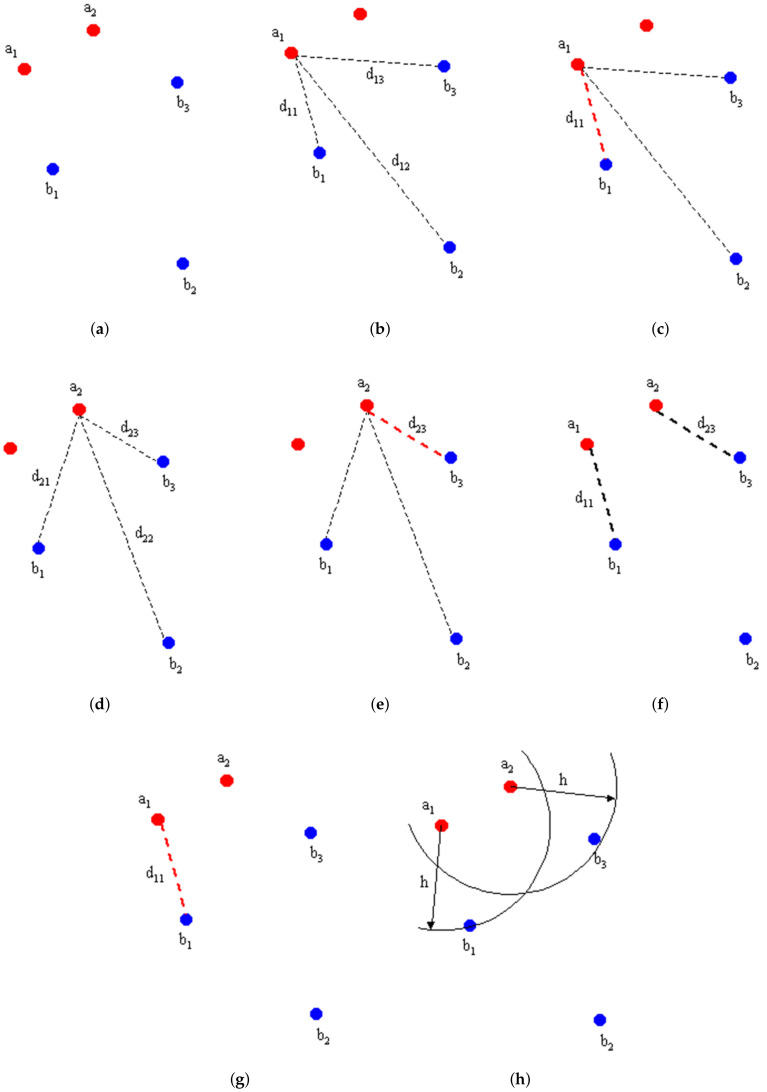
Algorithm flow chart. Reprinted with permission from [21]. Copyright 1998, copyright Normand Grégoire. (**a**) Define the set of points *A* and *B*. (**b**) Calculate $a_{1}$ and *B* set distances. (**c**) Obtain the shortest distance. (**d**) Calculate $a_{2}$ and *B* set distances. (**e**) Obtain the shortest distance. (**f**) Take the largest of the shortest distances. (**g**) Obtain h(A,B). (**h**) h(A,B) of the set of points *A* and *B*.

Assuming that *A* and *B* are two sets of points, a diagram of the procedure for calculating the Hausdorff distance algorithm is shown in Figure 7.

Given two sets of points *A* and *B*, we find the Hausdorff distance h(A,B) among them; then, we calculate the distances of all points in sets $a_{1}$ and *B* and select the shortest of them; again, we calculate the distances of all points in sets $a_{2}$ and *B* and select the shortest of them; then, we compare the shortest distances and take the maximum of them to be the Hausdorff distance.

**Figure 8:**
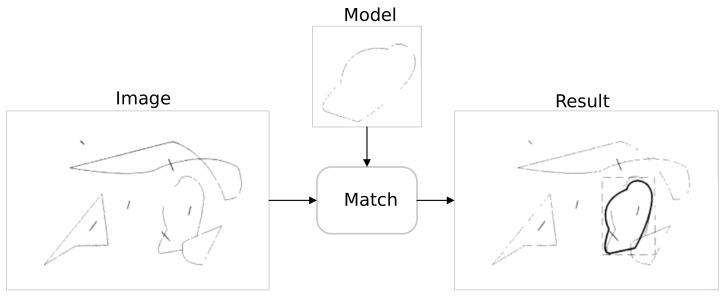
Hausdorf matching results [22].

Given a scene image and a target model, after performing matching through the Hausdorff distance, the matching result is as shown in Figure 8 below.

**Figure 9:**
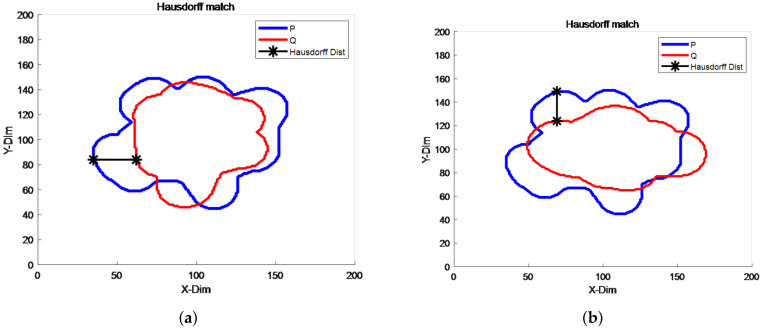
Graph of Hausdorff distance calculation results. (**a**) Data set P, Q calculation results. (**b**) Calculated results after Q change.

We are given two sets *P*, *Q* in MATLAB, where *P* is a fixed target set and *Q* takes a different target set; the maximum distance between the nearest points of the two sets is calculated after Hausdorff distance matching by imitating the late target vehicle signal and the radar target signal, the results of which are shown in Figure 9 below.

As can be seen in Figure 8, the Hausdorff distance matching algorithm can successfully calculate the Hausdorff distance for both sets, marked with a starred black line.

#### 3.1.2. Improved Hausdorff Distance Matching Algorithm

As the traditional Hausdorff distance matching algorithm only calculates the distance information characterized in the target data detected by the two sensors, the error in the results of calculating the degree of similarity between the two sensors using a single piece of distance information is large and the robustness of the system is not satisfactory. When two objects are particularly close to coinciding in their spatial position during target detection, the fusion system is unable to discriminate between the two, resulting in poor or incorrect detection results. This paper calculates the similarity of the targets jointly with the velocity information of the weighted targets in the traditional Hausdorff distance matching algorithm. If the deviation of the distance and velocity values of the targets detected by the two sensors is within the set threshold, the targets detected by the two sensors can be considered as the same object.

We define the position–velocity correlation matrix of the targets detected by the two sensors in the traditional way, denoted by ${\left\{D\right\}}_{m\times n}$, where $D_{ij}$ is an element in this matrix, to represent the position–velocity matching deviation between the ith camera target and the *j*th millimeter-wave radar detection target. Commonly used calculation methods are considered below.

(17) $$D_{ij}=\left|Cd_{i}-Rd_{j})\right|\times df+\left|Cv_{i}-Rv_{j}\right|\times vf+ClassT\left(CClass_{i},RClass_{j}\right)\times Classf$$

where $Cd_{i}$ denotes the distance measurement of the second *i*th target in the camera and $Rd_{j}$ denotes the distance measurement of the first *j*th target in the millimeter-wave radar. $Cv_{i}$ is the *i*th target whose velocity value is detected in the video image, $Rv_{j}$ is the *j*th target velocity value measured in the millimeter-wave radar, vf and df denote the deviation influence factors of distance and velocity, respectively, while the matrix related to the target type can be represented by ClassT. $CClass_{i}$ and $RClass_{j}$ denote the type of the *i*th target detected in the camera video and the *j*th target detected in the radar, respectively, and the influence factor of the deviation of the target type between the two is Classf.

The $\left|Cd_{i}-Rd_{j})\right|$ in the traditional calculation formula is the absolute distance difference between two target points in space, because the distance measurement values obtained directly from the camera and millimeter-wave radar sensors will contain a large number of errors, and some of the detected distance values are not vehicle target information. These perturbation errors will greatly affect the success of target matching and can easily cause false detection. The Hausdorff distance calculation results in taking all the distance data obtained by the two sensors, calculating the distance from each point in the millimeter-wave radar distance data set to all points in the camera distance set separately, selecting the shortest distance from each point in the millimeter-wave radar to the camera distance set, and finally comparing them to take the maximum value of these shortest distances as the distance deviation value between the two sensors. Compared with the absolute distance difference, the Hausdorff distance represents a distance defined between any two sets in the metric space, determined by the maximum value of the two distances, which measures the maximum mismatch between the two sets of points; thus, the matching deviation calculated by the Hausdorff distance is more accurate.

The Hausdorff distance value $H(C_{i},R_{j})$ between the *i*th camera video target and the *j*th millimeter-wave radar target is calculated by Equation (16) and brought into Equation (17) to obtain the improved deviation calculation formula shown in Equation (18) below.

(18) $$D_{ij}=\left|H(C_{i},R_{j})\right|\times df+\left|Cv_{i}-Rv_{j}\right|\times vf+ClassT\left(CClass_{i},RClass_{j}\right)\times Classf$$

#### 3.1.3. Millimeter-Wave Radar and Camera Target Matching

**Figure 10:**
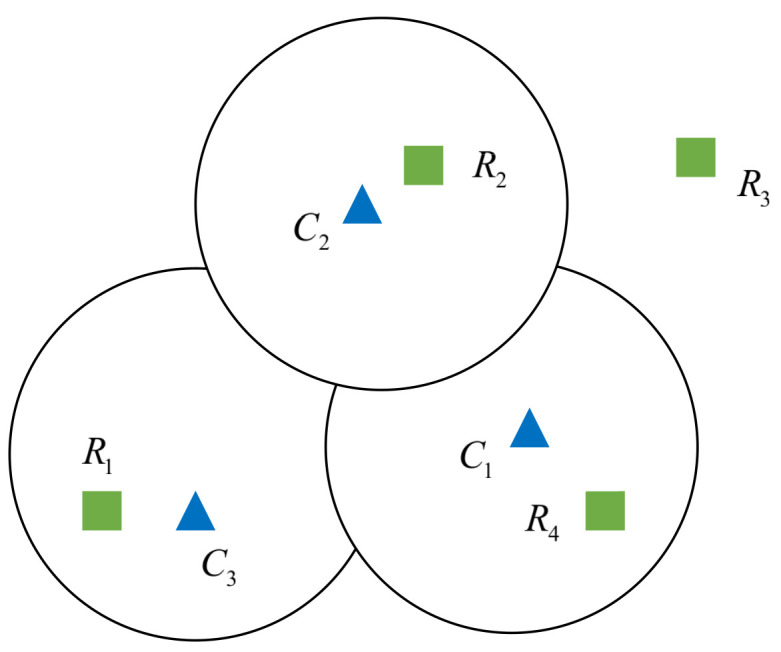
Camera and millimeter-wave radar detection of target planes.

Assume that, at any given moment, the camera detects *m* maneuvering targets in the video data and the millimeter-wave radar detects *n* targets. Due to certain differences in the detection performance of each sensor itself, the number of targets detected by the two sensors *m* and *n* is not exactly equal, and their detection schematic is shown in Figure 10. Assuming that the camera detects a target number *m* of 3 and the radar detects a target number *n* of 4, we denote the target vehicles detected in the camera video by $C_{1}$, $C_{2}$, and $C_{3}$, while the targets detected by the radar are denoted by $R_{1}$, $R_{2}$, $R_{3}$, and $R_{4}$.

Assuming that the deviation threshold for matching is *D*th, we calculate the deviation ${\left\{D\right\}}_{m\times n}$ of matching the *i*th target detected in the camera video with the *j*th target detected by the radar according to Equation (18). We find the data target pair that meets the condition $D_{ij}<D$th in the given matrix *D*th according to the deviation threshold ${\left\{D\right\}}_{m\times n}$, and then determine the data target pair that is characterized by the smallest deviation value $D_{ij}$ in the data target pair. If the *i*th target detected in the video acquired by the camera has the greatest similarity to the *j*th target in the target data detected by the radar, it can be decided that the data pair can represent the same object from which the two sensors detected the target, and the next step of target matching can be carried out to complete the target matching.

**Figure 11:**
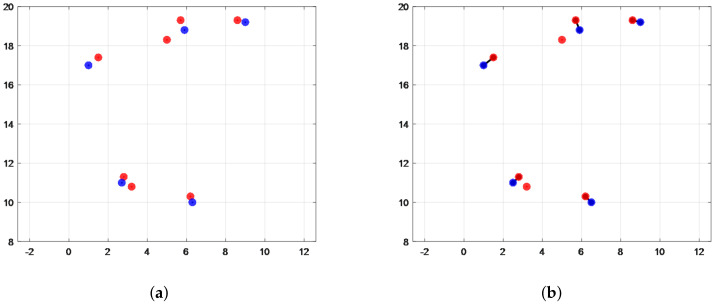
Target matching diagram. (**a**) Data pairs. (**b**) Matching results.

Given two sets, representing radar-detected data and video-detected target data, respectively, the matching relationship is illustrated by the matching relationship diagram in Figure 11, where the red dot represents the target point detected by the millimeter-wave radar, the blue dot represents the center point at the bottom edge of the rectangular box of the target detected by the image, and the black connecting line represents the matching connection relationship between the point sets formed by the data collected by the two sensors.

In Figure 11, the target data detected by the external environment interference radar are on the high side, but the radar and video data basically finish matching. The experimental results prove that the target matching algorithm proposed in the paper is realistic and effective.

### 3.2. Target Detection Models

**Figure 12:**
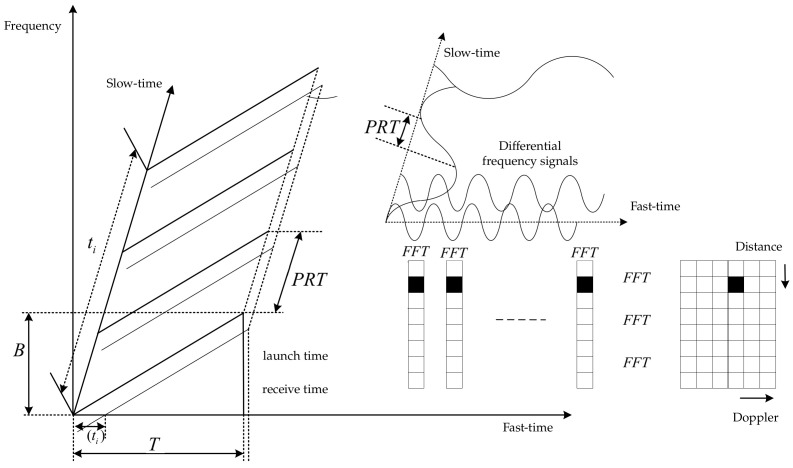
LFMCW two-dimensional measurement principle.

The paper maps the millimeter-wave radar-detected targets into the image coordinate system by means of coordinate transformation, and the detection results are presented as images. The video data acquired by the camera are used to separate the motion foreground using a hybrid Gaussian background modeling algorithm to extract the region of interest of the image. For radar sensors, linear frequency-modulated continuous wave is a special millimeter-wave technology. The LFMCW radar system obtains multiple sets of differential frequency signals after fast- and slow-time two-dimensional FFT operation, which can effectively control the false alarm rate and extract the distance and velocity information of the target, and the measurement principle is shown in Figure 12.

The target data collected by the camera are used to obtain the position and velocity information of the target by using the monocular visual velocimetry method proposed by Zhen Sang, and the linear regression model [23]. The origin of the camera coordinate system is the origin of the world coordinate system, and the conversion equation from the pixel coordinate feature point to the world coordinate system is established; the coordinates of the corresponding spatial feature point ${\left(X_{w},Y_{w},Z_{w}\right)}^{T}$ are obtained from the coordinates ${\left(u,v\right)}^{T}$ on the image, with the following equation:

(19) $$\left(\begin{array}{c} X_{w}\\ Y_{w}\\ Z_{w}\\ 1 \end{array}\right)=K\left(\begin{array}{c} u\\ v\\ 1 \end{array}\right)=\left(\begin{array}{c} k_{1}\;\;k_{2}\;\;k_{3}\\ k_{4}\;\;k_{5}\;\;k_{6}\\ k_{7}\;\;k_{8}\;\;k_{9}\\ k_{10}\;k_{11}\;k_{12} \end{array}\right)\left(\begin{array}{c} u\\ v\\ 1 \end{array}\right)$$

where $X_{w}$ is the vertical height of the target feature point in the world coordinate system, $Y_{w}$ is the lateral displacement of the target feature point relative to the origin of the coordinates, and $Z_{w}$ represents the vertical displacement between the target feature point and the world coordinate system. ${\left(k_{10},k_{11},k_{12}\right)}^{T}={\left(0,0,1\right)}^{T}$ in the mapping matrix *K*. The remaining nine unknown parameters can be derived from the mapping matrix *K* by establishing a monocular ranging model and measuring the corresponding coordinate values of multiple sets of feature points in the pixel coordinate system and the world coordinate system, according to which the actual displacement of the feature points can be derived from the mapping relationship, and the relative distance between the target vehicle and this vehicle can then be deduced. Solving the ranging equation is a process of finding the optimal solution to a linear system of equations, and the least squares derivation steps are as follows.

For a model with parameter $b_{1},\ldots ,b_{q}$ having multiple uncorrelated variables $x_{1},\ldots ,x_{q}$, the linear function is expressed as follows:

(20) $$y\left(x_{1},\ldots ,x_{q};b_{0}b_{1},\ldots ,b_{q}\right)=b_{0}+b_{1}x_{1}+\ldots +b_{q}x_{q}$$

which is expanded into a linear system of equations:

(21) $$\left\{\begin{array}{c} b_{0}+b_{1}x_{11}+\ldots +b_{j}x_{1j}+\ldots +b_{q}x_{1q}=y_{1}\\ b_{0}+b_{1}x_{21}+\ldots +b_{j}x_{2j}+\ldots +b_{q}x_{2q}=y_{2}\\ \vdots \\ b_{0}+b_{1}x_{i1}+\ldots +b_{j}x_{ij}+\ldots +b_{q}x_{iq}=y_{i}\\ \vdots \\ b_{0}+b_{1}x_{n1}+\ldots +b_{j}x_{nj}+\ldots +b_{q}x_{nq}=y_{n} \end{array}\right.$$

where *A* denotes the matrix consisting of $x_{ij}$, *b* denotes the matrix consisting of $b_{j}$, and *Y* is the observation $Y_{j}$. We then solve the system of linear equations.

(22) $$\left(\begin{array}{c} 1\;x_{11}\cdots x_{1j}\cdots x_{1q}\\ 1\;x_{21}\cdots x_{2j}\cdots x_{2q}\\ \vdots \;\vdots \;\cdots \;\vdots \;\cdots \;\vdots \\ 1\;x_{i1}\cdots x_{ij}\cdots x_{iq}\\ \vdots \;\vdots \;\cdots \;\vdots \;\cdots \;\vdots \\ 1\;x_{n1}\cdots x_{nj}\cdots x_{nq} \end{array}\right)\left(\begin{array}{c} b_{0}\\ b_{1}\\ \vdots \\ b_{j}\\ \vdots \\ b_{q} \end{array}\right)=\left(\begin{array}{c} y_{1}\\ y_{2}\\ \vdots \\ y_{i}\\ \vdots \\ y_{n} \end{array}\right)$$

that is, Ab=Y, and the least squares method with linear squared differences yields the result $\min_{b}\;{∥Ab=Y∥}_{2}$. The optimal solution is as follows:

(23) $$b={\left(A^{T}A\right)}^{-1}A^{T}Y$$

according to the least squares method, for Equation (22).

(24) $$\left(\begin{array}{c} X_{w}\\ Y_{w}\\ Z_{w}\\ 1 \end{array}\right)=K\left(\begin{array}{c} u\\ v\\ 1 \end{array}\right)=\left(\begin{array}{c} k_{1}k_{2}k_{3}\\ k_{4}k_{5}k_{6}\\ k_{7}k_{8}k_{9}\\ 001 \end{array}\right)\left(\begin{array}{c} u\\ v\\ 1 \end{array}\right)$$

the optimal solution is as follows:

(25) $$\left(\begin{array}{c} k_{1}\\ k_{2}\\ k_{3} \end{array}\right)={\left(A^{T}A\right)}^{-1}A^{T}B,\left(\begin{array}{c} k_{4}\\ k_{5}\\ k_{6} \end{array}\right)={\left(A^{T}A\right)}^{-1}A^{T}C,\left(\begin{array}{c} k_{7}\\ k_{8}\\ k_{9} \end{array}\right)={\left(A^{T}A\right)}^{-1}A^{T}D$$

of which

(26) $$A=\left(\begin{array}{c} u_{1}\;v_{1}\;1\\ u_{2}\;v_{2}\;1\\ \;\vdots \;\vdots \;\;\vdots \\ u_{i}\;v_{i}\;1\\ \;\vdots \;\vdots \;\;\vdots \\ u_{n}\;v_{n}\;1 \end{array}\right)$$

(27) $$B=\left(\begin{array}{c} X_{w1}\\ X_{w2}\\ \vdots \\ X_{wi}\\ \vdots \\ X_{wn} \end{array}\right),C=\left(\begin{array}{c} Y_{w1}\\ Y_{w2}\\ \vdots \\ Y_{wi}\\ \vdots \\ Y_{wn} \end{array}\right),D=\left(\begin{array}{c} Z_{w1}\\ Z_{w2}\\ \vdots \\ Z_{wi}\\ \vdots \\ Z_{wn} \end{array}\right)$$

on the basis of this model, the model was calibrated by real vehicle test trials to obtain the model parameter *K* values. In order to ensure that the ranging model is valid and universal, the calibration experiments were carried out by fixing the camera at a specified position, moving the vehicle target and keeping the target vehicle within 40 m of the sensor, and measuring the horizontal and vertical distance of the midpoint of the licence plate behind the vehicle relative to the center of the camera using a tape measure. Some of the calibration data are shown in Table 2.

The range coefficient matrix is obtained after fitting by least squares:

(28) $$K=\left(\begin{array}{c} 0\;\;0\;\;-115\\ 1.2931\;\;-0.2914\;-548.2838\\ 0\;\;\;\;-11\;\;10125\\ 0\;\;0\;\;\;1 \end{array}\right)$$

the paper is therefore based on a model of monocular visual ranging as follows:

(29) $$\left(\begin{array}{c} X_{w}\\ Y_{w}\\ Z_{w}\\ 1 \end{array}\right)=\left(\begin{array}{c} 0\;\;0\;\;-115\\ 1.2931\;\;-0.2914\;-548.2838\\ 0\;\;\;\;-11\;\;10125\\ 0\;\;0\;\;\;1 \end{array}\right)\left(\begin{array}{c} u\\ v\\ 1 \end{array}\right)$$

let the world coordinates of the target feature point of a frame be denoted by $M(X_{w},Y_{w},Z_{w})$, and after Δt time, the coordinates of the feature point in the next frame are denoted by $M(X_{w}^{*},Y_{w}^{*},Z_{w}^{*})$. Based on the operational relationship of the velocity displacement, the relative velocity of the detected target is solved as follows:

(30) $$\Delta \vec{v}=\frac{\sqrt{{\left(X_{w}-X_{w}^{*}\right)}^{2}+{\left(Y_{w}-Y_{w}^{*}\right)}^{2}+{\left(Z_{w}-Z_{w}^{*}\right)}^{2}}}{\Delta t}$$

The Gaussian mixture model proposed by Stauffer C [24] is used for video moving target detection, using the weighted sum of multiple Gaussian distributions to describe the distribution of each pixel in the image sequence, and, when a new frame is input, the model is updated using the previously updated mean, variance, and other parameters. The higher the number of Gaussian distributions, the more accurately the background image reflects the change in position of the target, and the acquired target motion area is closer to the true result, but the algorithm will also be more complex. When a new target appears, the Gaussian distribution is reordered using the ratio of weight to variance $\omega_{i,t}/\sigma_{i,t}^{2}$, and the *K* Gaussian distributions in the model are ordered according to the size of the ratio. Because the Gaussian distribution describing the background is located at the front of the sequence, the first *B* Gaussian distributions are selected as the background model and the remaining Gaussian distributions are the foreground model.

(31) $$B=\arg\min_{b}\left(\sum \begin{array}{c} b\\ i=1 \end{array}\omega_{i,t}>T\right)$$

where *T* is the threshold value, which is taken to be 0.65 according to the test.

## 4. Test Results and Analysis

**Figure 13:**
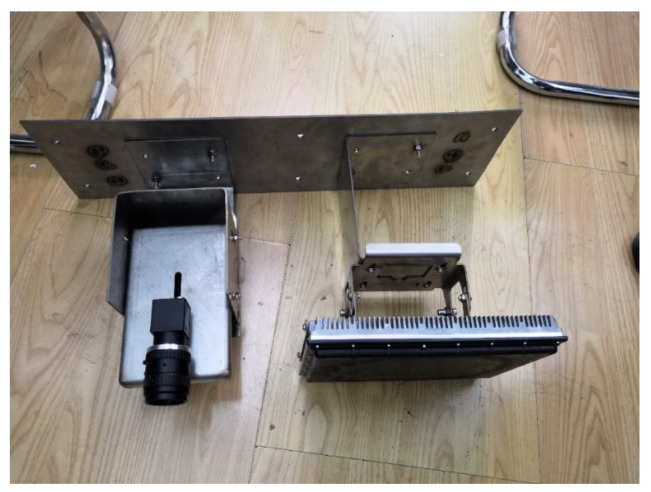
Physical view of the sensor.

In order to better verify the effectiveness of the above proposed vehicle detection method, the millimeter-wave radar used in this paper is the SMS series UMRR-0A model from Smartmicro, Germany, and the camera is the Hikvision DS-2CD2D25DWD model, representing the fusion of the two sensors, as shown in Figure 13, with the camera on the left and the millimeter-wave radar on the right.

### 4.1. Building a Real-World Experimental Platform

When building the experimental platform, the pitch angle of the radar is determined in accordance with the actual requirements of use. When the radar is at a fixed installation height, the radar is in the vertical observation area at different elevation angles. The installation height and the elevation angle of the radar have a great influence on the optimum operating conditions and the best detection distance of the radar. The influence of mounting height, azimuth, tilt, and horizontal angle on the detection accuracy should be taken into account when the millimeter-wave radar is actually installed. Table 3 shows the minimum detection distances obtained for radar sensors with different combinations of mounting height and elevation angle.

**Figure 14:**
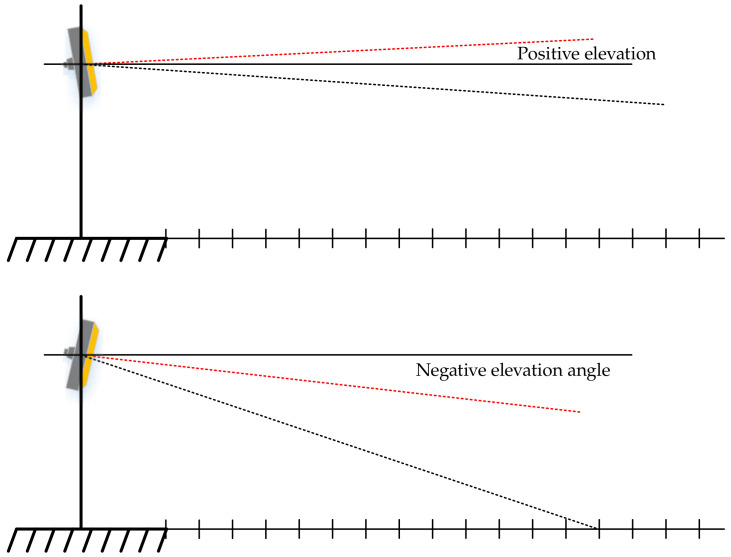
Positive and negative elevation angles for millimeter-wave radar sensors.

The SMS millimeter-wave radar is limited by the minimum detection distance, which is influenced by the installation height and angle. When collecting data, the experimental equipment was installed on a gantry approximately 6 m above the ground, so the best detection results can be obtained by adjusting the radar installation angle according to the actual requirements. The installation angle of the radar is differentiated between positive and negative elevation angles, and the detection distances for positive and negative elevation angles are not the same. The installation angle of the millimeter-wave radar is shown schematically in Figure 14 below, The red line represents the actual installation angle, and the black line represents the detection angle.

**Figure 15:**
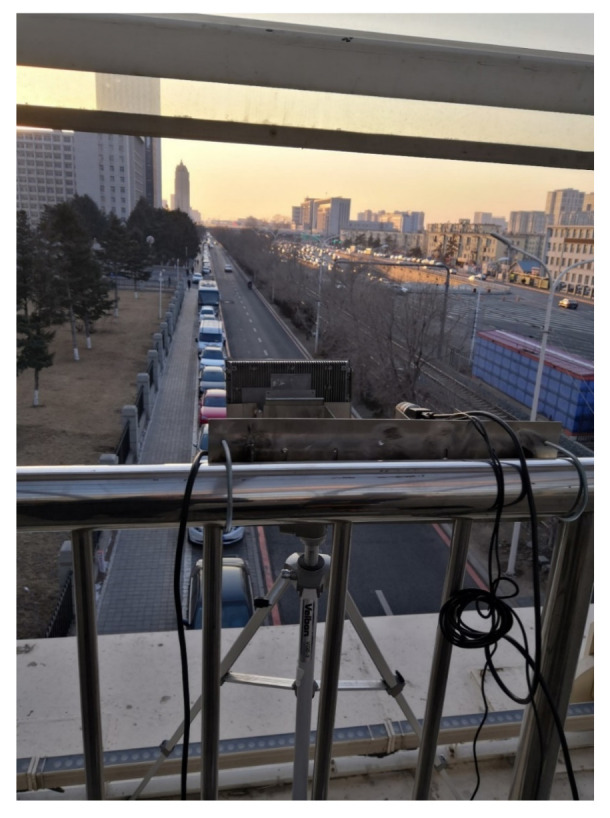
Millimeter-wave radar and camera experimental scene installation diagram.

It is worth noting that a negative elevation angle is suitable for short-distance detection; a positive elevation angle is suitable for uphill roads. Due to the flat surface of the experimental environment and the short detection distance of the camera, this experiment takes a negative elevation angle approach, i.e., a 3° downward tilt installation, in order to ensure that the azimuth of the radar points to the area of the lane to be detected. The camera is mounted in close proximity to the millimeter-wave device and can be spatially approximated to the same physical coordinates. When the millimeter-wave radar and camera were fused for the experiment, a multi-sensor fusion experimental platform was built for data acquisition, and the actual installation scenario of the two sensors is shown in Figure 15.

Figure 15 shows the actual road scene selected for the fusion experiment scenario, with stationary vehicles on both sides of the road. The video target extraction algorithm can filter out the effect of stationary vehicles on the detection results.

### 4.2. Data Acquisition and Processing Platform

**Figure 16:**
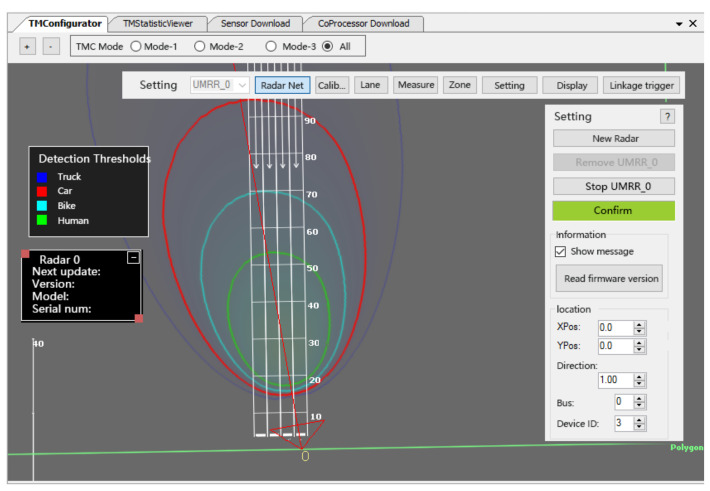
TMC software interface.

The millimeter-wave radar data processing environment is the TMC software. Based on the millimeter-wave radar data reception parsing algorithm to interpret the target position and velocity information detected by the millimeter-wave radar, this paper uses the UMRR radar configuration software TMC to process the millimeter-wave radar data; the millimeter-wave radar data processing software interface is shown in Figure 16. In the actual road monitoring system, the ID number of the targets acquired by the millimeter-wave radar for the numbering order is independent of the distance of the target from the sensor and depends only on the order in which they are detected in the scene.

Figure 16 shows that the radar data processing software can monitor not only different types of vehicles, but also pedestrians and bicycles on the road, making the monitoring range particularly broad and suitable for basically all traffic monitoring scenarios.

**Figure 17:**
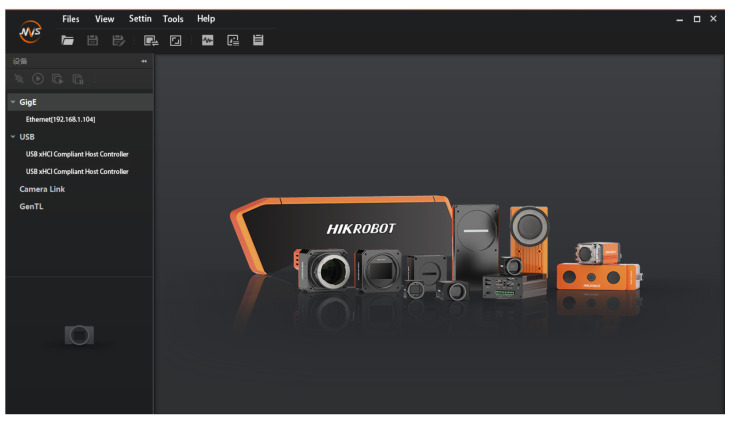
MVS software interface.

The camera capture data environment is the official downloadable MVS software from Hikvision’s HIKROBOT, which saves the captured video stream to a local path and processes the video data through the hybrid Gaussian background modeling algorithm described in Section 3 to extract regions of interest. The MVS software interface is shown in Figure 17.

### 4.3. Video Motion Target Extraction

**Figure 18:**
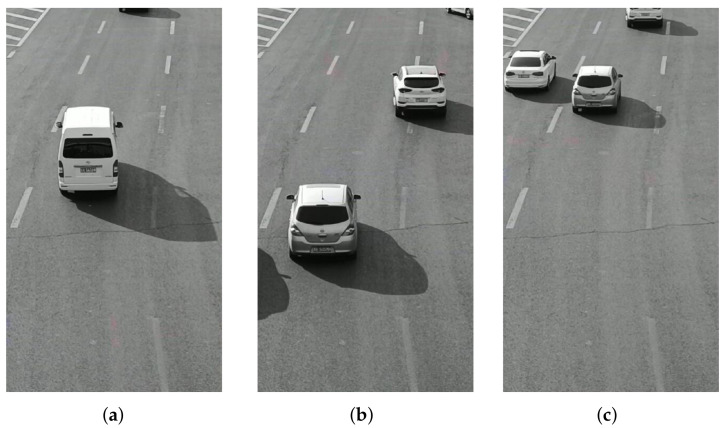
The original video sequence. (**a**) Frame 11. (**b**) Frame 61. (**c**) Frame 141.

**Figure 19:**
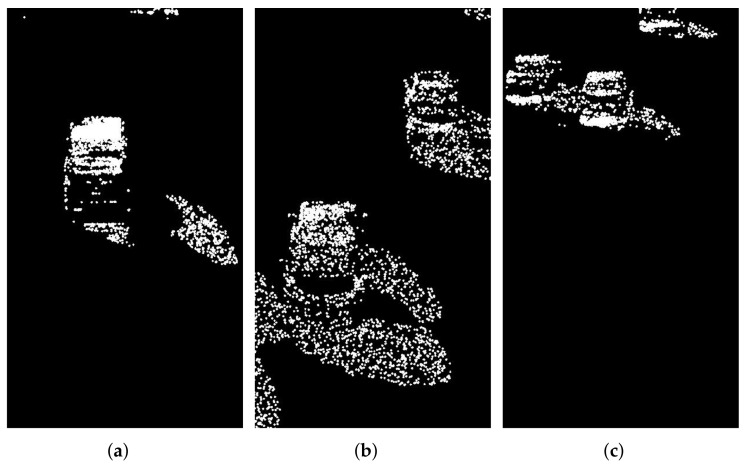
MOG processing. (**a**) Frame 11. (**b**) Frame 61. (**c**) Frame 141.

**Figure 20:**
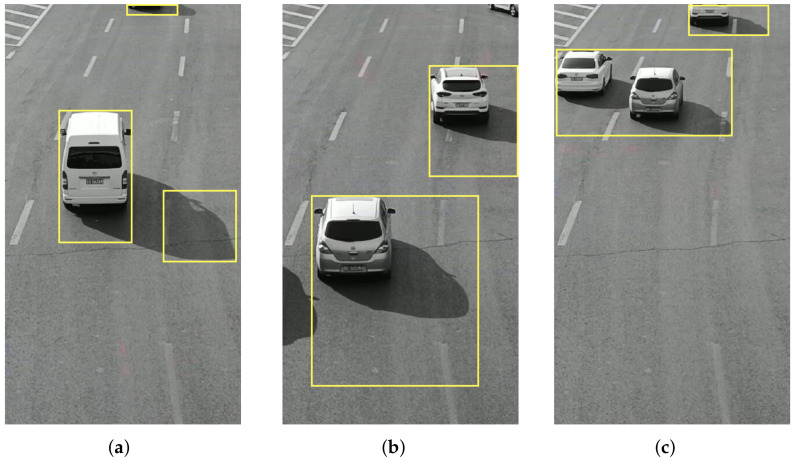
Video region of interest. (**a**) Frame 11. (**b**) Frame 61. (**c**) Frame 141.

The video data processing environment is MATLAB2018a software; its programming environment consists of a series of software tools, and the call class library is rich, so when processing video data, one can perform mixed programming in MATLAB for experiments. According to the mixed Gaussian background modeling algorithm described in Section 3, from a set of video sequences, we randomly extracted 3 frames of images for testing, and set a mixed Gaussian number of 3, after several experiments. The learning rate of the algorithm was finally determined to be 0.025 and the foreground threshold was 0.65. The experimental results are included as follows: Figure 18 shows the 11th, 61st, and 141st frames of the video sequence extracted, respectively; Figure 19 shows the result of the hybrid Gaussian background modeling process; Figure 20 shows the region of interest formed by the video after the hybrid Gaussian background modeling, marked with a yellow rectangular box.

From Figure 19, it can be seen that for the vehicle target detected by the video after the hybrid Gaussian background modeling algorithm, due to the problem of light, the shadow of the body of the vehicle in the process of driving also changes with the movement of the target vehicle, resulting in the hybrid Gaussian detected target containing the projection of the vehicle, and therefore in the target region of interest formed in the video, causing the target to avoid the false detection situation. In Figure 20, the 11th frame of the image shows a false detection of the vehicle shadow situation, and two targets are detected for the same target vehicle, while the 141st frame of the image is not ideal for single video detection as the two vehicles are too close together during the driving process and are detected as the same target by the video moving target detection system, resulting in a missed detection situation.

For the monocular vision ranging algorithm using a data regression-based model, shown in Figure 20, it is assumed that the world coordinate system of the experimental vehicle regression frame feature point is ${\left(X_{w},Y_{w},Z_{w}\right)}^{T}$, and $X_{w}$ is the vertical displacement of the feature point from the camera center, which is not calculated when ranging, and the relative distance of the vehicle can be obtained according to the horizontal and vertical coordinate distance of the vehicle in Equation (31). The results are retained to two decimal places.

(32) $$L=\sqrt{{Y_{w}}^{2}+{Z_{w}}^{2}}$$

the distance of the target in the rectangular box in Figure 20 was calculated using the velocimetric distance measurement model. A total of seven vehicle targets were detected in the video image and the actual distance was measured using a tape measure; the comparative distance measurement results are shown in Table 4.

As can be seen from the table, the distance measurement model based on the data regression frame is more accurate for vehicle distance measurement in the range of 0–30 m, with an error of less than 2%, and the calculation error in the range of 30–40 m is less than 5%, with an average error of 1.87%, which indicates high distance measurement accuracy.

The corresponding image frames in Figure 20 are frame 11, frame 61, and frame 141. The distance change of the moving object is obtained according to the change between two frames, and the motion speed of the target is obtained by combining the motion time difference of adjacent image frames. Since the vertical displacement $X_{w}$ of the feature point from the camera center is a fixed value, the speed measurement formula can be simplified as follows.

(33) $$\Delta \vec{v}=\frac{\Delta x}{\Delta t}=\frac{\sqrt{{\left(Y_{w}-Y_{w}^{*}\right)}^{2}+{\left(Z_{w}-Z_{w}^{*}\right)}^{2}}}{\Delta t}$$

During the frame splitting process, the time difference between two adjacent frames is calculated to be 0.35 s, and the speed measurement results are shown in Table 5 below.

In Table 4 and Table 5, the distance velocity values of the first two targets are similar because they are the same vehicle and the video is misdetected as two targets due to vehicle shadowing.

### 4.4. Multi-Sensor Fusion Experiments

**Figure 21:**
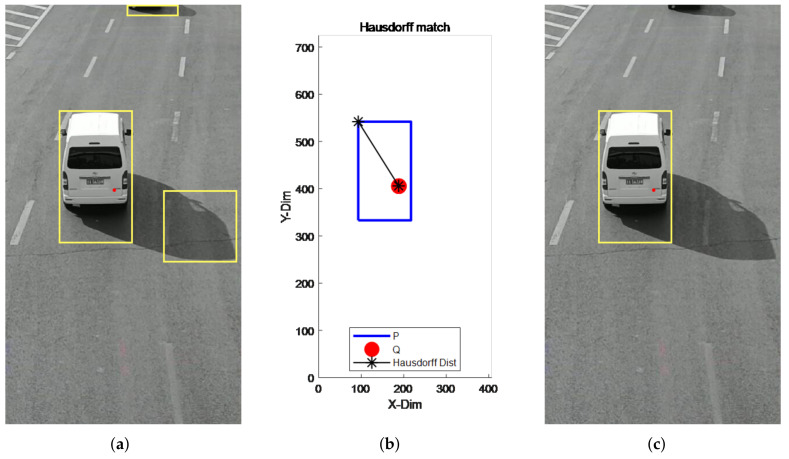
Hausdorff distance matching results of radar and video. (**a**) Pre-treatment. (**b**) Hausdorff match. (**c**) Post-treatment.

For the case of video false detection and missed detection, the target detected by the millimeter-wave radar was fused and matched with the video to jointly achieve target detection. The improved Hausdorff distance matching algorithm was used, based on the traditional distance matching algorithm, incorporating the target velocity value. By comparing the calculated matching deviation with the set threshold, we determined whether the two were the same target, and verified whether the target detected by the video was the real target, with the target information detected by the millimeter-wave radar. The detection results are shown in Figure 21 below.

**Figure 22:**
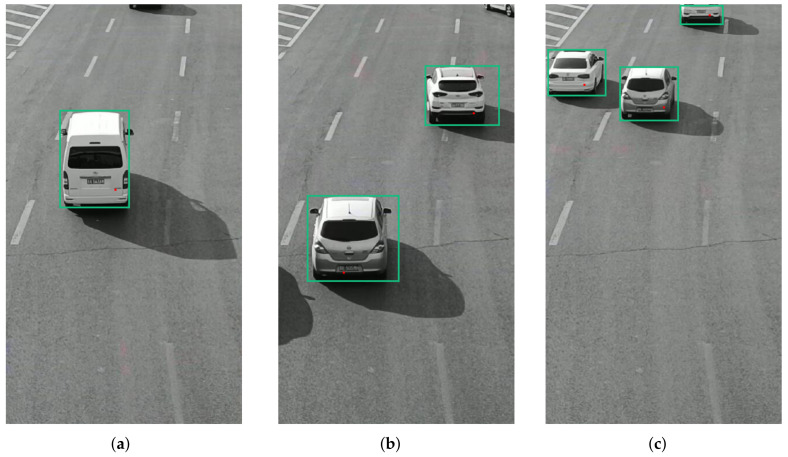
Radar and video fusion detection results. (**a**) Frame 11. (**b**) Frame 61. (**c**) Frame 141.

As can be seen from Figure 21, the Hausdorff distance matching algorithm can effectively filter out the false detection targets in the video by eliminating the interference caused by external lighting. After the matching of the two sensor targets was completed, the successfully matched target data were subjected to target detection, the experimental results of which are shown in Figure 22. The red dots in the figure are the results of detection using a single millimeter-wave radar, and the fusion detection results are marked by the green rectangular boxes.

By comparing the detection results in Figure 20 and Figure 22, it can be seen that the moving target vehicles in the region of interest can be detected using a single camera sensor, but the detection results do not achieve the expected results, and there are even cases of false target detection and missed detection. The fusion system can accurately detect all moving vehicles in the region of interest, reducing the impact of environmental factors on the experimental results, which can be seen as a significant improvement in the accuracy of the fusion detection results.

### 4.5. Experimental Verification on Public Datasets

In order to prove the effectiveness of the method proposed in this paper, the public road monitoring data set UA-DETRAC is introduced and compared with the existing traditional method of calculating deviation value matching. It is proven that the method proposed in this paper is effective and can accurately locate the matching target in the case of occlusion. We selected one of the sets of experimental results as follows.

**Figure 23:**
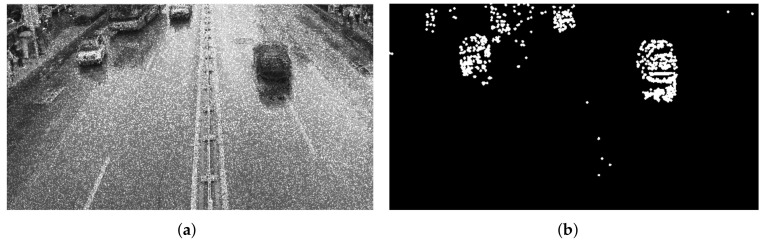
Mixed Gaussian processing. (**a**) Gaussian blend background image. (**b**) Gaussian mixture foreground image.

**Figure 24:**
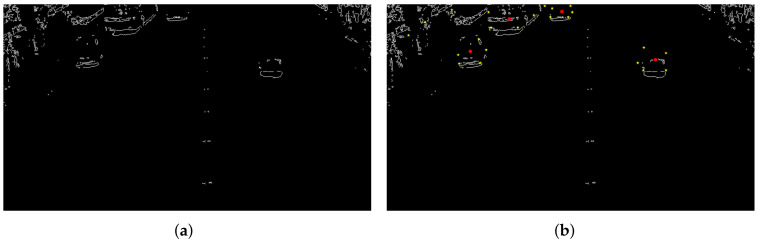
Match ID result. (**a**) Image contour extraction. (**b**) Combined radar signals.

As can be seen in Figure 23, the Gaussian mixture process can control the region of interest by varying the threshold. Moving objects are covered, but objects that are too close or objects affected by light can still be confused. Figure 24 below is an annotation of the radar monitoring results on the contour processing map. The yellow point is the aggregation area of radar points, and the red point is a schematic point for the convenience of identifying the target selection.

Since the process of radar point cloud accumulation can take time, generally, only 3–5 points are calculated to identify the target. It can be seen that the accuracy of the final result after the radar point aggregation position and the vehicle contour are matched is very high.

**Figure 25:**
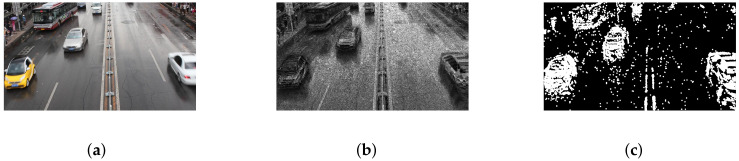
Direct extraction. (**a**) Original image. (**b**) Gaussian blend background image. (**c**) Final extracted image.

**Figure 26:**
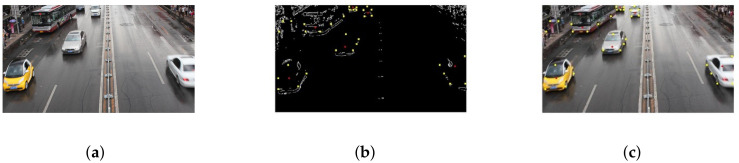
Improved Hausdorff matching. (**a**) Original image. (**b**) Improved Hausdorff matching. (**c**) Final extracted image.

The selected MVI65352 data have a total of 985 frames, of which there are 30 handover frames, similar to the above figure. Using the traditional matching method, the data of the vehicles that are occluded by each other are lost, as shown in Figure 25. With the matching method in this paper, the vehicles are all detected correctly, as shown in Figure 26. The following is a comparison of the two test results.

It can be seen from the figure that in the case of occlusion, the point cloud aggregation signal of the radar signal can be matched with the image-processed signal by the Hausdorff distance, thereby improving the matching success rate. With the aid of the radar velocity component, better results can be obtained.

## 5. Discussion

This paper addresses the problem of poor recognition alignment due to mutual occlusion between vehicles and external environmental perturbations in intelligent transportation systems. Based on spatio-temporal alignment, millimeter-wave radar data are mapped to images to form a radar region of interest, while hybrid Gaussian background modeling is used to separate motion foregrounds to obtain an image region of interest. The Hausdorff distance matching algorithm proposed in the paper is used to perform target matching between the millimeter-wave radar and the video region of interest to determine whether the two measurements are descriptions of the same target vehicle. Finally, the successfully matched data are used for target vehicle identification, thus completing vehicle detection. The experimental results show that the fusion detection results can overcome the problem of a low video detection rate when the vehicle has shadows and occlusions. Compared with the measurement results of a single sensor with a similar cost, and the traditional matching method of directly calculating the deviation value, the method proposed in this paper has higher robustness and better performance in the case of shading, shadows, and light effects in road monitoring test results.

## Figures and Tables

**Table 1:** Comparison table of sensor performance.

Performance	Ultrasound	Infrared	LIDAR	Vision	Millimeter-Wave Radar
Long-range detection	−1	0	1	1	1
Target identification	−1	−1	0	1	1
Exclusion of false alarms	−1	−1	0	0	1
Temperature stability	−1	0	1	1	1
Climate impact	0	1	1	1	0
Darkness penetration	1	1	1	0	1
Hardware costs	−1	−1	0	1	1

**Table 2:** Calibration data collected.

No.	World Coordinate System	Pixel Coordinate System
1	(−115, 0, 380)	(639.26, 942.71)
2	(−115, 0, 1528)	(641.21, 780.86)
3	(−115, 0, 2206)	(632.47, 763.23)
4	(−115, −292, 858)	(369.7, 842.17)
5	(−115, −292, 858)	(369.7, 842.17)
6	(−115, 243, 2488)	(723.96, 756.39)

**Table 3:** Minimum detection distance.

Elevation Angle/°												
		−5	−4	−3	−2	−1	0	1	2	3	4	5
	10	32.71	34.87	37.32	40.11	43.31	47.05	51.45	56.71	63.14	71.15	81.44
	9	29.44	31.39	33.59	36.10	38.98	42.34	46.30	51.04	56.82	64.04	73.30
	8	26.17	27.90	29.86	32.09	34.65	37.64	41.16	45.37	50.51	56.92	65.15
	7	22.90	24.41	26.12	28.08	30.32	32.93	36.01	39.70	44.20	49.81	57.01
H/m	6	19.63	20.92	22.39	24.06	25.99	28.23	30.87	34.03	37.88	42.69	48.87
	5	16.35	17.44	18.66	20.05	21.66	23.52	25.72	28.36	31.57	35.58	40.72
	4	13.08	13.95	14.93	16.04	17.33	18.82	20.58	22.69	25.26	28.46	32.58
	3	9.81	10.46	11.20	12.03	12.99	14.11	15.43	17.01	18.94	21.35	24.43
	2	6.54	6.97	7.46	8.02	8.66	9.41	10.29	11.39	12.63	14.23	16.29
	1	3.27	3.49	3.73	4.01	4.33	4.70	5.14	5.67	6.31	7.12	8.14

**Table 4:** Distance measurement results.

Actual Measuring Distance (m)	Model Calculated Distance (m)	Error (%)
20.21	20.12	0.44
19.35	19.11	1.24
41.52	39.86	4.00
18.36	18.11	1.36
30.27	30.05	0.77
35.68	34.79	2.49
40.59	39.45	2.81

**Table 5:** Velocity measurement results.

Δx (m)	Δt (m)	Speed (m/s)
8.89	0.35	25.40
8.91	0.35	25.46
12.48	0.35	35.67
11.65	0.35	33.28
14.11	0.35	40.31
15.83	0.35	45.23
13.85	0.35	39.58
